# Molecular regulation of lung maturation in near-term fetal sheep by maternal daily vitamin C treatment in late gestation

**DOI:** 10.1038/s41390-021-01489-4

**Published:** 2021-04-15

**Authors:** Erin V. McGillick, Sandra Orgeig, Beth J. Allison, Kirsty L. Brain, Youguo Niu, Nozomi Itani, Katie L. Skeffington, Andrew D. Kane, Emilio A. Herrera, Janna L. Morrison, Dino A. Giussani

**Affiliations:** 1 Early Origins of Adult Health Research Group, Health and Biomedical Innovation, University of South Australia, Adelaide, SA Australia; 2 Molecular and Evolutionary Physiology of the Lung Laboratory, UniSA: Clinical and Health Sciences, University of South Australia, Adelaide, SA Australia; 3 Department of Physiology, Development and Neuroscience, University of Cambridge, Cambridgeshire, UK; 4 Programa de Fisiopatología, Instituto de Ciencias Biomédicas, Facultad de Medicina, Universidad de Chile, Santiago, Chile; 5 Cambridge BHF Centre of Research Excellence, University of Cambridge, Cambridgeshire, UK; 6 Cambridge Strategic Research Initiative in Reproduction, University of Cambridge, Cambridgeshire, UK

## Abstract

**Background:**

In the fetus, the appropriate balance of prooxidants and antioxidants is essential to negate the detrimental effects of oxidative stress on lung maturation. Antioxidants improve respiratory function in postnatal life and adulthood. However, the outcomes and biological mechanisms of antioxidant action in the fetal lung are unknown.

**Methods:**

We investigated the effect of maternal daily vitamin C treatment (200 mg/kg, intravenously) for a month in late gestation (105–138 days gestation, term ~145 days) on molecular regulation of fetal lung maturation in sheep. Expression of genes and proteins regulating lung development was quantified in fetal lung tissue. The number of surfactant-producing cells was determined by immunohistochemistry.

**Results:**

Maternal vitamin C treatment increased fetal lung gene expression of the antioxidant enzyme *SOD-1*, hypoxia signaling genes (*HIF-2α*, *HIF-3α*, *ADM*, and *EGLN-3*), genes regulating sodium movement (*SCNN1-A*, *SCNN1-B*, *ATP1-A1*, and *ATP1-B1*), surfactant maturation (*SFTP-B* and *ABCA3*), and airway remodeling (*ELN*). There was no effect of maternal vitamin C treatment on the expression of protein markers evaluated or on the number of surfactant protein-producing cells in fetal lung tissue.

**Conclusions:**

Maternal vitamin C treatment in the last third of pregnancy in sheep acts at the molecular level to increase the expression of genes that are important for fetal lung maturation in a healthy pregnancy.

**Impact:**

- Maternal daily vitamin C treatment for a month in late gestation in sheep increases the expression of gene-regulating pathways that are essential for normal fetal lung development.
- Following late gestation vitamin C exposure in a healthy pregnancy, an increase in lung gene but not protein expression may act as a mechanism to aid in the preparation for exposure to the air-breathing environment after birth.
- In the future, the availability/development of compounds with greater antioxidant properties than vitamin C or more specific targets at the site of oxidative stress in vivo may translate clinically to improve respiratory outcomes in complicated pregnancies at birth.

## Introduction

Antioxidants play a vital role in promoting health by scavenging free radicals and preventing oxidative stress.^1^ Healthy pregnancy itself is associated with a pro-oxidant state leading to increased lipid peroxidation.^2–4^ In addition, there is an increased fetal demand for antioxidants during the rapid phase of fetal growth in the last 15% of gestation.^5^ Therefore, an adequate maternal antioxidant defense to protect the fetus from oxidative stress is essential for normal development.

An imbalance between a pro- and antioxidant status is associated with a wide range of pregnancy complications, including intrauterine growth restriction, preeclampsia, hypertension, and gestational diabetes, as well as with environmental factors, such as maternal smoking, all of which can contribute to respiratory complications at birth.^1,3,6–10^ Importantly, it has been suggested that neonatal complications like respiratory distress may result from the effects of oxidative stress on the molecular regulation of lung maturation in utero.^8^ Antioxidants have been shown to improve lung function in children of mothers who smoked in pregnancy^11^ and as a result of supplementation in adulthood.^12,13^ However, research on the effects of oxidative stress or of antioxidant treatment on fetal lung maturation has been scant in either healthy or complicated pregnancy.

In a healthy pregnancy, the development of the fetal endogenous antioxidant system in late gestation parallels the maturation of several mechanisms that regulate lung development.^5^ These include the maturation of the surfactant system and of processes regulating lung liquid movement with advancing gestation, including those triggered by glucocorticoids and by hypoxia.^5,14–16^ This is important because oxidized surfactant proteins (SPs) show reduced surface tension and immune regulation that may predispose to newborn respiratory distress.^17–21^ Moreover, the regulation of fetal lung liquid reabsorption and the maintenance of the epithelial fluid lining in the lung are both important, as lung fluid has a role in the pulmonary defense to oxidative stress at the air–liquid interface.^22,23^ Combined, therefore, past data highlight the importance of endogenous antioxidants in normal development and protecting the development of molecular pathways that regulate fetal lung maturation, thereby enabling the successful transition to air breathing at birth.^1^

However, whether maternal antioxidant supplementation to this endogenous antioxidant defense in a healthy pregnancy is beneficial or detrimental to the molecular regulation of fetal lung maturation is completely unknown. Therefore, in this study, we tested the hypothesis that maternal treatment with vitamin C in healthy pregnancy has beneficial effects on the molecular regulation of fetal lung maturation. The hypothesis was tested in sheep, as this species has a temporal profile of lung maturation similar to humans.^24^ Understanding the effect of antioxidants on the fetal lung in normal development is an important step towards possible antioxidant therapy to promote fetal lung maturation in complicated pregnancy.

## Methods

All procedures were approved by the University of Cambridge Ethical Review Board and were performed in accordance with the UK Animals (Scientific Procedures) Act 1986.

### Surgery and experimental protocol

At 100 ± 1 days gestation (term, ~145 days), 17 pregnant Welsh mountain ewes carrying singleton pregnancies underwent a sterile laparotomy under general anesthesia (1.5–2.0% isofluorane in 60:40 O_2_:N_2_O) to determine fetal sex and catheterization of the maternal femoral artery and vein, as previously described.^25–27^ To control for sex differences, only male fetuses were included in this study (female fetuses were assigned to a postnatal study). At 105 days gestation (when the fetal lung is in the canalicular phase of development), ewes were randomly assigned to one of two experimental groups: receiving either daily bolus intravenous saline (*n* = 8) or vitamin C (200 mg/kg intravenously (i.v.) daily; *n* = 9) between 105 and 138 days gestation. Vitamin C was chosen for administration in this study due to it being a commonly used water-soluble antioxidant supplement that can cross the placenta^28^ and shows powerful antioxidant protection in the offspring.^27,29–31^ While vitamin C can be easily administered orally daily in humans, the maternal i.v. route was chosen in this study to ensure complete and better controlled delivery into the maternal circulation. Maternal blood gases, hemoglobin concentration and saturation were recorded during baseline (104 and 105 days), 1 day following treatment (106 days), and then as summary averages of the preceding 5 days for 110, 115, 120, 125, 130, and 135 days gestation. Blood gas and acid–base values were measured using an ABL5 blood gas analyzer (Radiometer; Copenhagen, Denmark). Values for percentage saturation of hemoglobin with oxygen and for the concentration of hemoglobin in blood were determined using a hemoximeter (OSM3; Radiometer).^25,26^ At these time intervals, a maternal blood sample was collected for subsequent analysis of vitamin C concentration in plasma.

### Post mortem and sample collection

Fetuses were evaluated near term at 138 days gestation when the lung is in the alveolar stage of development similar to human late preterm birth (36–37 weeks of gestation; term = 40 weeks^24^). All ewes and their fetuses were killed by an overdose of sodium pentobarbitone (0.4 mL/kg, intravenous administration, Pentoject; Animal Ltd, York, UK) and fetuses were delivered by hysterotomy. As the fetuses in this study were not catheterized, a fetal umbilical arterial blood sample was taken at post mortem for measurement of plasma vitamin C and cortisol concentrations. Fetal body and organ weights were recorded. The tissues generated in this study were part of a program of work designed with a different primary objective.^27^ This study used the tissues generated to begin to address additional important scientific questions retrospectively, thereby making the best use of the valuable experimental material. This scientific approach is strongly promoted by the UK Home Office 3R principle of Replacement, Reduction, and Refinement designed to ensure more humane animal research. Consequently, no prospective functional outcomes were performed and the lung tissue collected was immersion fixed rather than perfusion fixed. A piece of left fetal lung tissue was snap frozen in liquid nitrogen and stored at −80 °C for gene and protein expression analysis. A section of right fetal lung tissue was immersion fixed in 4% paraformaldehyde and processed to paraffin for further immunohistochemical analysis.

### Maternal blood gas status and maternal and fetal plasma assays

Plasma concentrations of vitamin C were measured by a fluorimetric technique using a centrifugal analyzer with a fluorescence attachment, according to the method of Vuilleumier and Keck,^32^ in collaboration with the Core Biochemical Assay Laboratory (Cambridge, UK). The inter-assay coefficients of variation were 7.9% at 27.1 µmol/L and 5.0% at 89.7 µmol/L, the lower limit of detection of the assay was 10 µmol/L. Plasma cortisol concentrations in fetal blood collected were measured using a commercially available ELISA kit (RE52061, IBL International, Germany), according to the manufacturer’s guidelines as previously described.^15,25^

### Quantification of fetal lung messenger RNA (mRNA) expression

RNA was extracted and complementary DNA synthesized from fetal lung tissue samples (~50 mg; saline, *n* = 8; vitamin C, *n* = 9) as previously described.^15,33^ The expression of target genes (Table 1) regulating oxidative stress, antioxidant defenses, hypoxia signaling, glucocorticoid signaling, lung liquid secretion and reabsorption (regulated by chloride, sodium, and water transport across the pulmonary epithelium), surfactant maturation (protein and lipid synthesis), and airway remodeling were measured by quantitative reverse transcription-polymerase chain reaction as previously described.^15^ The abundance of each transcript relative to the abundance of stable reference genes (β-actin, peptidylprolyl isomerase, tyrosine 3-monooxygenase) was calculated using DataAssist 3.0 analysis software and is expressed as mRNA mean normalized expression ± SEM.^15^

**Table 1:** Evaluation of target genes regulating oxidative stress, hypoxia signaling, glucocorticoid signaling, fetal lung liquid movement (controlled by chloride, sodium, and water movement), surfactant maturation, and airway remodeling by quantitative real-time RT-PCR (all primer sequences and concentrations previously published^15^).

	Gene name	Protein name	Function
Oxidative stress			
Nicotinamide adenine dinucleotide phosphate oxidase	*NOX-4*	NAPDH oxidase 4	Pro-oxidant marker
Heme oxygenase-1	*HMOX-1*	HMOX-1	Pro-oxidant marker
Inducible nitric oxide synthase	*NOS-2*	iNOS	Pro-oxidant marker
Endothelial nitric oxide synthase	*NOS-3*	eNOS	Pro-oxidant marker
Superoxide dismutase enzymes	*SOD-1*	SOD-1	Antioxidant marker
	*SOD-2*	SOD-2	
Catalase	*CAT*	CAT	Antioxidant marker
Glutathione peroxidase	*GPX*	GPx	Antioxidant marker
Hypoxia signaling			
Hypoxia-inducible factor subunits	*HIF-1α*	HIF-1α	Major regulator of hypoxia signaling
	*HIF-2α*	HIF-2α	
	*HIF-3α*	HIF-3α	
	*HIF-1β*	HIF-1β	
Vascular endothelial growth factor	*VEGF*	VEGF	Hypoxia-responsive gene
Adrenomedullin	*ADM*	ADM	Hypoxia-responsive gene
Lysine (K)-specific demethylase 3 A	*KDM3A*	JMJD1A	Hypoxia-responsive gene
Solute carrier family 2 (facilitated glucose transporter) member 1	*SLC2A1*	GLUT-1	Hypoxia-responsive gene
Egl-9 family hypoxia-inducible factor enzymes (encoding the prolyl hydroxylase domain proteins)	*EGLN-1*	PHD-2	Regulator of HIF activity and signaling
	*EGLN-2*	PHD-1	
	*EGLN-3*	PHD-3	
Glucocorticoid signaling			
11β-hydroxysteroid dehydrogenase enzyme -1	*HSD11B-1*	11βHSD-1	Glucocorticoid-activating enzyme isoform
11β-hydroxysteroid dehydrogenase enzyme -2	*HSD11B-2*	11βHSD-2	Glucorticoid-deactivating enzyme isoform
Glucocorticoid receptor	*NR3C1*	GR	Cellular glucocorticoid receptor
Mineralocorticoid receptor	*NR3C2*	MR	Cellular glucocorticoid receptor
Molecular regulation of lung liquid movement			
Cystic fibrosis transmembrane conductance regulator	*CFTR*	CFTR	Chloride transport channel on pulmonary epithelium
Chloride channel voltage-sensitive 2 channel	*CLCN2*	CLC2	Chloride transport channel on pulmonary epithelium
Epithelial sodium channel subunits	*SCNN1-A*	ENAC-α	Sodium transport channel on pulmonary epithelium
	*SCNN1-B*	ENAC-β	
	*SCNN1-G*	ENAC-γ	
Sodium potassium adenosine triphosphatase subunits	*ATP1-A1*	Na-K-ATPase-α1	Sodium transport channel on pulmonary epithelium
	*ATP1-B1*	Na-K-ATPase-β1	
Aquaporin	*AQP-1*	AQP-1	Channels regulating water movement across pulmonary epithelium
	*AQP-3*	AQP-3	
	*AQP-4*	AQP-4	
	*AQP-5*	AQP-5	
Surfactant maturation and lipid transport			
Surfactant protein	S*FTP-A*	SP-A	Involved in pulmonary immunity
	S*FTP-B*	SP-B	Involved in regulating surface tension
	S*FTP-C*	SP-C	Involved in regulating surface tension
	S*FTP-D*	SP-D	Involved in pulmonary immunity
Phosphate cytidylyltransferase 1, choline, alpha	*PCYT1A*	PCYT1A	Surfactant lipid synthesis
ATP-binding cassette, sub-family A (ABC1), member 3	*ABCA3*	ATP-A3	Surfactant lipid transport
Airway remodeling			
Elastin	*ELN*	ELN	Structural role in lung tissue development
Collagen type 1 alpha 1	*COL1A1*	COL	Structural role in lung tissue development

### Quantification of fetal lung protein expression

Protein was extracted by sonication of fetal lung tissue (~100 mg; saline, *n* = 6; vitamin C, *n* = 7) and protein content determined by a MicroBCA Protein Assay Kit (Pierce, Thermo Fisher Scientific Inc., Rockford, IL) as previously described.^34^ Extracted protein samples (75–100 μg) were subject to sodium dodecyl surface (SDS) page and stained with Coomassie blue to determine equal protein loading. Protein samples were transferred onto a 0.45 µm nitrocellulose membrane (Hybond ECL, GE HealthCare, NSW, Australia), subjected to 1 h of drying at room temperature, and then stained with Ponceau S (0.5% Ponceau in 1% acetic acid) in order to determine the efficacy of the transfer. The membranes were briefly washed with 7% acetic acid followed by a reverse osmosis water rinse, and then imaged for Ponceau S using ImageQuant LAS4000 (GE Healthcare, Victoria, Australia). Following imaging, membranes were washed 3 × 5 min in tris-buffered saline (TBS). The membranes were blocked in 5% bovine serum albumin (BSA) in TBS with 1% Tween-20 (TBS-T) for 1 h at room temperature. The membranes underwent 3 × 5 minutes washes in TBS-T and were incubated with the primary antibody overnight at 4 °C (ENAC-β (1:1000, in 5% BSA in TBS-T, #PA5-77817, Invitrogen; 87 kDa band); Na^+^-K^+^-ATPase-A1 (1:1000 in 5% BSA in TBS-T, #MA3-929, Invitrogen; 110 kDa), Na^+^-K^+^-ATPase-B1 (1:1000 in 5% BSA in TBS-T, #MA3-930, Thermo Fisher; 50 kDa band); 11βHSD-2 (1:1000, in 5% BSA in TBS-T, #10004303, Cayman Chemical; 44 kDa band); SOD-1 (1:1000, in 5% BSA in TBS-T, #A3854, Sigma Aldrich; 24 kDa band); SP-B (1:1000, in 5% BSA in TBS-T, #WRAB-48604, Seven Hills Bioreagents; 8 kDa band)). The primary antibodies were chosen based on genes that changed in response to maternal vitamin C administration in the fetal lung to determine if the transcriptional changes observed translated into protein abundance differences. Following incubation with the primary antibody, the blots were washed and incubated with the relevant species of horseradish peroxidase (HRP)-labeled secondary IgG antibody for 1 h at room temperature. Enhanced chemiluminescence using SuperSignal West Pico Chemiluminescent Substrate (Thermo Scientific, Australia) was used to detect the blots. The Western blot was imaged using ImageQuant LAS4000 and the protein abundance was quantified by densitometry using ImageQuant software (GE Healthcare, Victoria, Australia). Total target protein abundance was then normalized to total protein (Ponceau S) or to a reference protein, β-actin (1:10,000 in 5% BSA in TBS-T, ATCB HRP conjugate, #4967, Cell Signaling Technology; 42 kDa band), β-tubulin (1:10,000 in 5% BSA in TBS-T, β-tubulin (9F3) HRP conjugate, #5346, Cell Signaling Technology; 55 kDa band), or COX1V (3E11) (1:10,000 in 5% BSA in TBS-T, COX1V (3E11) HRP conjugate, #5247 P, Cell Signaling Technology; 17 kDa band).

### Quantification of surfactant-producing cells within the fetal lung

To determine the effect of maternal vitamin C administration on the surfactant-producing capacity of the fetal lung at the structural level, immunohistochemistry was performed (saline, *n* = 6; vitamin C, *n* = 9), using a rabbit anti-human mature SP-B antibody (1:500, WRAB-48604, Seven Hills Bioreagents, OH), as previously described.^15^ Sections were examined using Visiopharm new Computer-Assisted Stereological Toolbox (NewCAST) software (Visiopharm, Hoersholm, Denmark), and point counting was used to determine the numerical density of SFTP-B-positive cells present in the alveolar epithelium of fetal lung tissue, as previously described.^15,35^

### Statistical analyses

All statistical analyses were performed using Statistical Package for Social Sciences (SPSS) v20.1 (Chicago). Values for maternal blood gas status and plasma ascorbic acid concentrations were averaged over the sampling period. All data were evaluated for outliers ±2 SD from the mean for each treatment group. Comparison of maternal vitamin C concentrations between baseline and end of treatment between groups was determined using a two-way analysis of variance with Tukey’s post hoc test. All other data were compared using the Student’s *t* test for unpaired data (saline vs. vitamin C). All data are presented as mean ± SEM. For all comparisons, *P* < 0.05 was considered statistically significant.

## Results

### Maternal and fetal plasma vitamin C, maternal blood gas status, and fetal growth

Maternal basal arterial plasma vitamin C concentrations did not differ between groups (38.0 + 3.9; vitamin C: 43.8 + 3.3 μmol/L). While maternal arterial plasma vitamin C levels remained unchanged from baseline in control ewes (39.1 + 4.3 μmol/L), treatment with vitamin C significantly increased vitamin C plasma concentration in maternal plasma by the end of the experimental period (81.4 + 12.8 μmol/L; *P* < 0.05). Plasma levels of vitamin C measured in the sample taken from the fetal umbilical artery at post mortem were also elevated in fetuses from mothers treated with vitamin C compared to those treated with vehicle (Table 2). There was no significant effect of maternal vitamin C treatment on maternal arterial blood gases, pH, or hemoglobin oxygen saturation during the experimental period (Table 2). Similarly, there was no significant effect of maternal vitamin C treatment on fetal body weight, ratio of bi-parietal diameter to lower hind limb length, relative brain weight, or relative lung weight (Table 2).

**Table 2:** Effect of vitamin C treatment on mother and fetus.

	Saline (*n* = 8)	Vitamin C (*n* = 9)
Maternal pH (arbitrary units)	7.50 ± 0.01	7.50 ± 0.02
Maternal PaCO_2_ (mm Hg)	33.3 ± 0.6	34.0 ± 0.1
Maternal PaO_2_ (mm Hg)	104.2 ± 1.2	104.2 ± 1.0
Maternal Hb saturation (%)	103.6 ± 0.2	104 ± 0.2
Fetal body weight (kg)	3.99 ± 0.14	3.74 ± 0.35
Fetal ratio of bi-parietal diameter to hind limb lower length	3.69 ± 0.13	3.38 ± 0.12
Fetal relative brain weight (g/kg)	10.93 ± 0.19	12.34 ± 0.73
Fetal relative lung weight (g/kg)	26.05 ± 1.49	22.29 ± 1.26
Cord plasma vitamin C (μmol/L)	20.2 ± 1.2	30.1 ± 1.4*
Cord plasma cortisol (ng/mL)	17.6 ± 3.0	28.5 ± 6.2

Data are expressed as mean ± SEM. Data were analyzed by the Student’s unpaired *t* test. Maternal blood gas results are the average of samples collected during the experiential period. The fetal measurements results were collected at post mortem.

*P < 0.05 was considered significant.

### Expression of genes regulating oxidative stress

**Fig. 1:**
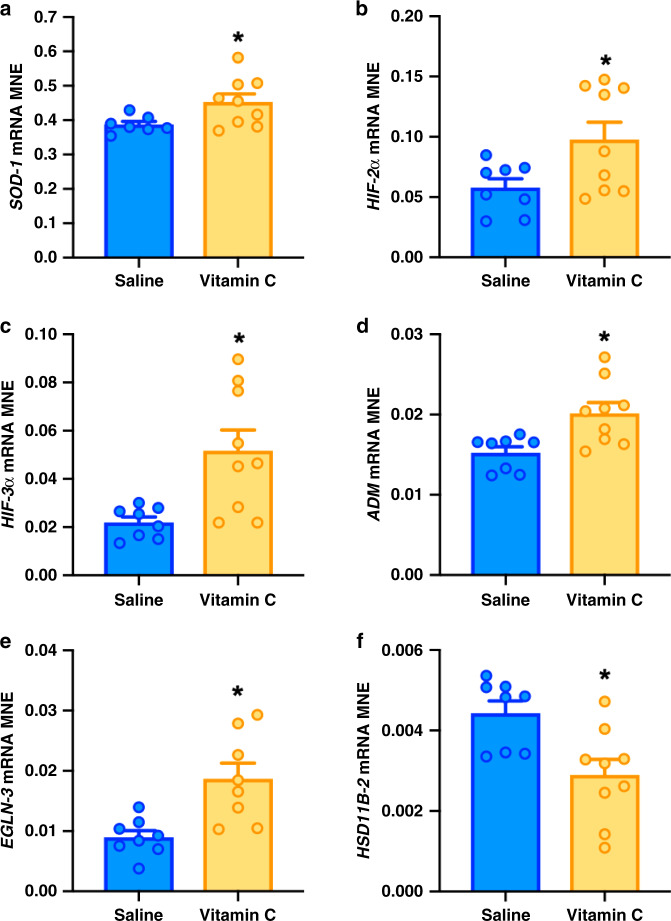
Expression of genes regulating antioxidant defence, hypoxia signaling and glucocorticoid availability. Effect of maternal vitamin C on expression of genes regulating antioxidant defence (*SOD-1*, **a**), hypoxia signaling (*HIF-2α*, **b**; *HIF-3α*, **c**; *ADM*, **d**; *EGLN-3*, **e**) and glucocorticoid availability (*HSD11B-2*, **f**) in the lung of the late gestation sheep fetus. Data are expressed as mRNA mean normalized expression (MNE) ± SEM in saline (blue bars, *n* =8) and vitamin C (orange bars, *n* =9) groups. Data were analyzed by the Student’s unpaired *t* test. *P < 0.05 was considered statistically significant.

There was no effect of maternal vitamin C treatment on the fetal lung mRNA expression of the pro-oxidant genes *NOX-4*, *HMOX-1*, *NOS-2*, or *NOS-3* (Table 3). However, maternal vitamin C treatment increased the fetal lung mRNA expression of the antioxidant *SOD-1* (Fig. 1a), but had no effect on the expression of *SOD-2*, *CAT*, or *GPX* (Table 3).

**Table 3:** Effect of maternal vitamin C treatment on expression of genes regulating oxidative stress, hypoxia signaling, glucocorticoid signaling, fetal lung liquid movement (controlled by chloride, sodium, and water movement), surfactant maturation, and airway remodeling in the fetal lung.

	Saline (*n* = 8)	Vitamin C (*n* = 9)
Pro-oxidant markers		
* NOX-4*	0.005 ± 0.001	0.004 ± 0.001
* HMOX-1*	0.029 ± 0.004	0.032 ± 0.003
* NOS-2*	0.019 ± 0.001	0.022 ± 0.002
* NOS-3*	0.007 ± 0.001	0.006 ± 0.0003
Antioxidant markers		
* SOD*-2	0.036 ± 0.004	0.042 ± 0.003
* CAT*	0.16 ± 0.01	0.20 ± 0.02
* GPX*	0.010 ± 0.002	0.017 ± 0.003
Hypoxia signaling		
* HIF-1α*	0.038 ± 0.002	0.042 ± 0.003
* HIF-1β*	0.036 ± 0.003	0.039 ± 0.002
* VEGF*	0.13 ± 0.01	0.16 ± 0.01
* KDM3A*	0.053 ± 0.001	0.059 ± 0.003
* SLC2A1*	0.011 ± 0.001	0.013 ± 0.001
* EGLN-1* (PHD-2)	0.062 ± 0.003	0.062 ± 0.004
* EGLN-2* (PHD-1)	0.022 ± 0.001	0.022 ± 0.001
Glucocorticoid signaling		
* HSD11B-1*	0.006 ± 0.001	0.007 ± 0.0004
* NR3C1*	0.15 ± 0.01	0.17 ± 0.01
* NR3C2*	0.007 ± 0.001	0.008 ± 0.001
Chloride transport		
* CFTR*	0.0040 ± 0.0002	0.0039 ± 0.0004
* CLCN2*	0.0034 ± 0.0003	0.0034 ± 0.0003
Sodium transport		
* SCNN1-G*	0.008 ± 0.002	0.015 ± 0.003
Water transport		
* AQP-1*	0.22 ± 0.01	0.23 ± 0.02
* AQP-3*	0.0013 ± 0.0002	0.0013 ± 0.0002
* AQP-4*	0.009 ± 0.002	0.012 ± 0.002
* AQP-5*	0.031 ± 0.003	0.038 ± 0.004
Surfactant maturation and lipid transport		
* SFTP-A*	0.43 ± 0.06	0.65 ± 0.11
* SFTP-C*	3.44 ± 0.40	4.14 ± 0.41
* SFTP-D*	0.029 ± 0.004	0.036 ± 0.006
* PCYT1A*	0.026 ± 0.002	0.024 ± 0.001
Airway remodeling		
* COL1A1*	1.44 ± 0.28	1.48 ± 0.23

Data are expressed as mean normalized expression ± SEM. Data were analyzed by the Student’s unpaired *t* test. *P* < 0.05 was considered significant.

### Expression of genes regulating hypoxia signaling and feedback

There was increased mRNA expression of *HIF-2α* and *HIF-3α* (Fig. 1b, c), but no effect on *HIF-1α* or the constitutively expressed *HIF-1β* subunit (Table 3) in the lung of fetuses following maternal vitamin C treatment compared to the saline group. There was a significant increase in the expression of the hypoxia-responsive gene *ADM* (Fig. 1d) in the vitamin C group, but no impact on the expression of other hypoxia-responsive genes evaluated (*VEGF*, *KDM3A*, or *SLC2A1*; Table 3). There was no difference in the lung mRNA expression of the genes coding for the prolyl hydroxylase enzyme isoforms, PHD-2 and PHD-1, *EGLN-1*, or *EGLN-2* (Table 3), respectively. However, there was increased expression of the gene *EGLN-3* coding for the hypoxia signaling feedback factor, PHD-3, in the vitamin C group (Fig. 1e).

### Plasma cortisol and expression of genes regulating glucocorticoid availability and activity

There was no significant effect of maternal vitamin C treatment on fetal plasma cortisol concentration at the conclusion of the experiment (Table 2). There was reduced mRNA expression of glucocorticoid-deactivating enzyme *HSD11β-2* in the fetal lung following maternal vitamin C treatment (Fig. 1f). There was no effect of maternal vitamin C on glucocorticoid-activating enzyme *HSD11β-1* or receptors for downstream glucocorticoid signaling in the fetal lung (*NR3C1* and *NR3C2*; Table 3).

### Expression of genes regulating fetal lung liquid secretion and reabsorption

**Fig. 2:**
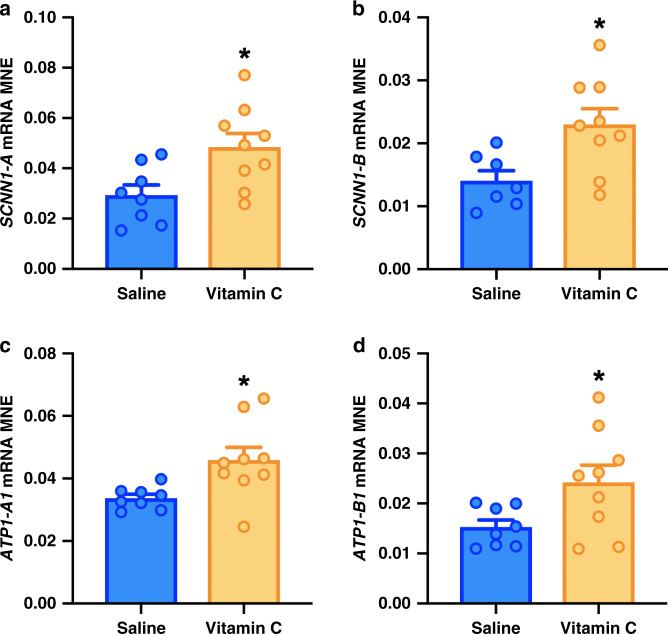
Expression of genes regulating sodium movement in the fetal lung. Effect of maternal vitamin C administration on the expression of genes regulating sodium movement (*SCNN1-A*, **a**; *SCNN1-B*, **b**; *ATP1-A1*, **c**; *ATP1-B1*, **d**) in the lung of the late gestation sheep fetus. Data are expressed as mRNA mean normalized expression (MNE) ± SEM in saline (blue bars, *n* = 8) and vitamin C (orange bars, *n* = 9) groups. Data were analyzed by the Student’s unpaired *t* test. *P < 0.05 was considered statistically significant.

There was no difference in the expression of genes regulating chloride movement (*CFTR* or *CLCN2*) between the saline and vitamin C group (Table 3). However, there was a significant increase in the expression of genes regulating sodium movement including *SCNN1-A*, *SCNN1-B*, *ATP1-A1*, and *ATP1-B1* subunits (Fig. 2a–d), but no effect on *SCNN1-G* mRNA expression (Table 3) in the vitamin C group compared to theh saline group. There was no difference in mRNA expression of aquaporin water transporter genes *AQP-1*, *AQP-3*, *AQP-4*, or *AQP-5* in the fetal lung (Table 3).

### Expression of genes regulating surfactant maturation and airway remodeling in the fetal lung

**Fig. 3:**
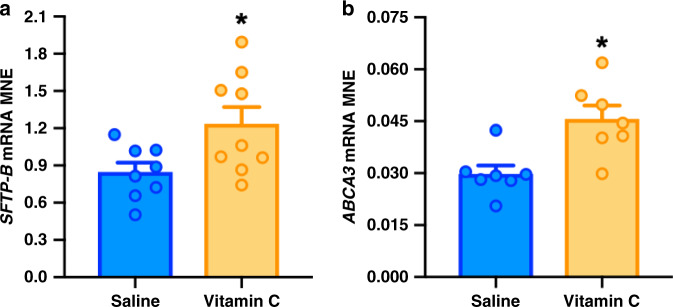
Expression of genes regulating surfactant maturation and lipid transport. Effect of maternal vitamin C administration on the expression of genes regulating surfactant maturation (*SFTP-B*, **a**) and surfactant lipid transport (*ABCA3*, **b**) in the lung of the late gestation sheep fetus. Data are expressed as mRNA mean normalized expression (MNE) ± SEM in saline (blue bars, *n* = 8) and vitamin C (orange bars, *n* = 9) groups. Data were analyzed by the Student’s unpaired *t* test. *P < 0.05 was considered statistically significant.

There was an increase in mRNA expression of the surfactant protein marker *SFTP-B* and the surfactant lipid transporter *ABCA3* in fetal lungs from the vitamin C compared to the saline group (Fig. 3a, b). However, there was no difference in the mRNA expression of fetal lung *SFTP-A*, *SFTP-C*, *SFTP-D* or surfactant phospholipid synthesis gene *PCYT1A* between groups (Table 3).

### Protein expression of genes that changed in response to maternal vitamin C administration

**Fig. 4:**
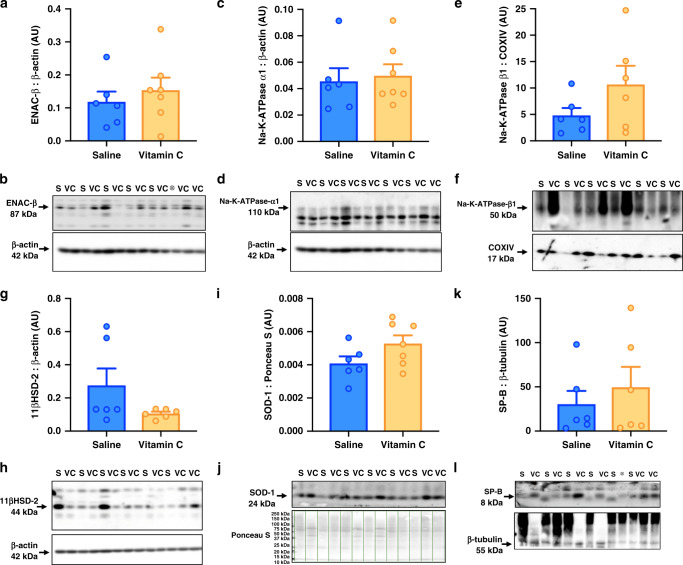
Protein expression for genes that changed in the fetal lung in response to maternal vitamin C administration. Data are presented as normalized protein expression in arbitrary units (AU) for ENAC-β (**a**; 87 kDa band), Na-K-ATPase α1 (**c**; 110 kDa band), Na-K-ATPase β1 (**e**; 50 kDa band), 11βHSD-2 (**g**; 44 kDa band), SOD-1 (**i**; 24 kDa band), and SP-B (**k**; 8 kDa band) in saline (blue bars) and vitamin C (orange bars) groups. Data were analyzed by the Student’s unpaired *t* test. *P* < 0.05 was considered statistically significant. Western blot images represent target protein (upper panel) and reference protein (lower panel) for lambs in saline (S) and vitamin C (VC) groups. ⊗ = Not included in the analysis for this study. Beta-actin (β-actin; **b**, **d**, **h**; 42 kDa band), cytochrome oxidase IV (COXIV; **e**; 17 kDa band), Ponceau S (**j**; Total protein), and β-tubulin (β-tubulin; **l**; 55 kDa band) are obtained from the same gel.

Despite changes in gene expression for the panel of markers investigated, there was no significant effect of maternal vitamin C on the expression of proteins (Fig. 4) involved in lung liquid reabsorption (ENAC-β, Na-K-ATPase-α1, Na-K-ATPase-β1), glucocorticoid activity (11βHSD-2), antioxidant status (SOD-1), or surfactant maturation (SP-B).

### Markers of structural development in the fetal lung

**Fig. 5:**
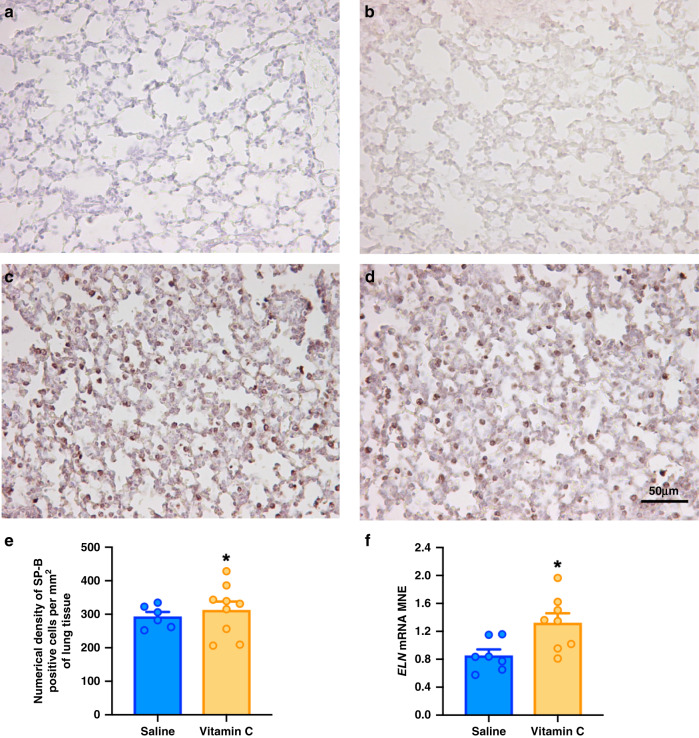
Evaluation of lung structure. Effect of maternal vitamin C administration on numerical density of SP-B-positive cells in the alveolar epithelium of immersion fixed fetal lung tissue (**a**–**e**) and expression of gene-regulating airway remodeling (*ELN* (**f**)). Data are expressed as mean or mRNA mean normalized expression (MNE) ± SEM in saline (blue bars, *n* = 8) and vitamin C (orange bars, *n* = 9) groups. Data were analyzed by the Student’s unpaired *t* test. *P < 0.05 was considered statistically significant. Micrographs demonstrating no primary antibody negative control (**a**), 1:500 rabbit serum negative control (**b**), SP-B immunoreactivity (brown intracellular precipitate) in the alveolar epithelium of the fetal lung following maternal saline (**c**) and vitamin C (**d**) administration for a month in late gestation. There was no significant effect of maternal vitamin C (orange bar, *n* = 8, **e**) on the numerical density of SP-B-positive cells per mm^2^ of lung tissue in the alveolar epithelium when compared to the saline fetal lung (blue bar, *n* = 6, **e**). Scale bar = 50 μm.

There was no effect of maternal vitamin C treatment on the numerical density of SP-B-positive cells present in the alveolar epithelium of the fetal lung tissue (Fig. 5a–e). There was increased fetal lung mRNA expression of *ELN* (Fig. 5f) following maternal vitamin C treatment; however, there was no effect on expression of *COL1A1* (Table 3).

## Discussion

The data show that maternal treatment with vitamin C for a month in late gestation in healthy pregnancy in sheep increases the expression of genes regulating antioxidant defenses (*SOD-1*), sodium movement (*SCNN1-A*, *SCNN1-B*, *ATP1-A1*, and *ATP1-B1*), surfactant maturation (*SFTP-B* and *ABCA3*), and airway remodeling (*ELN*) in the fetal lung. These effects of maternal vitamin C supplementation occurred in parallel with an increased expression of genes regulating hypoxia signaling (*HIF-2α*, *HIF-3α*, *ADM*, and *EGLN-3*). Conversely, there was no effect of maternal vitamin C on fetal plasma cortisol, or on the expression of glucocorticoid receptors in the fetal lung, or on the number of surfactant protein-producing cells in fetal lung tissue, or on the expression of protein markers evaluated. Therefore, the data support the hypothesis tested and suggest that maternal treatment with vitamin C in healthy pregnancy in sheep promotes the molecular regulation of fetal lung maturation by increasing gene expression via cortisol-independent pathways.

There was a significant effect of maternal vitamin C treatment on the expression of genes regulating hypoxia signaling. This included an increase in the expression of the *HIF-a* subunit, a key regulator of normal fetal lung development^36^ and surfactant maturation.^15,37,38^ Due to the relatively short half-life of the HIF-α protein subunits, we investigated the expression of the *HIF-*α gene and of genes with hypoxia-responsive elements in their promoter region, including *ADM* and *EGLN-3*. Regulation of hypoxia signaling is controlled by HIF-α subunit stability, which is negatively regulated by the prolyl hydroxylase domain (PHD) family of enzymes encoded by the EGLN gene (*EGLN-1*/PHD-2, *EGLN-2*/PHD-1, and *EGLN-3*/PHD-3).^16,39,40^ Following maternal vitamin C treatment, there was evidence for hypoxia signaling feedback by increased *EGLN-3* expression. Interestingly, there are similar changes in the lung and heart of the chronically hypoxemic and growth-restricted fetus.^15,16,37,38,41^ Therefore, this may be an adaptive mechanism to limit hypoxia signaling following exposure to pro-oxidant *milieux*, such as chronic fetal hypoxia associated with complicated pregnancy or the relative hyperoxia at birth in normal pregnancy.^42,43^ As vitamin C or ascorbate is a cofactor for the PHD enzyme activity,^44^ the increased *EGLN-3* expression (gene encoding PHD-3) suggests that there may also be antioxidant-independent effects of vitamin C on the regulation of hypoxia signaling in the fetal lung in late gestation.

The effect of either endogenous or exogenous antioxidants on the molecular regulation of fetal lung liquid movement has not been previously investigated. This study provides evidence for a limited effect of maternal vitamin C treatment on the expression of genes regulating chloride movement, which is a molecular regulator of active liquid secretion into the developing lung throughout gestation.^24,45^ Conversely, there was increased gene expression of subunits regulating sodium movement, which is important in controlling fetal lung liquid active reabsorption before birth and basal regulation of liquid movement at the air–liquid interface in the air-breathing lung.^24,46–48^ On balance, we did not observe a significant effect of maternal vitamin C treatment on the expression of the panel of proteins investigated that play a role in the molecular regulation of lung liquid movement. There was a positive effect of maternal vitamin C on the expression of genes regulating surfactant maturation and surfactant lipid transport. Taken together, these data suggest that at the genomic level maternal vitamin C treatment promotes the expression of molecular markers that aid in the transition to air breathing at birth; however, these have not translated into significant effects on protein expression. The non-coordinate regulation of gene and protein expression observed in this study may be due to several possibilities. For instance, discordant regulation of gene and protein expression may be due to differences in post-transcriptomic modification or transcript degradation following fetal exposure to antenatal vitamin C. Alternatively, it may simply represent that at the time that tissue was collected these signals were not being actively translated as the system had already been maximally upregulated. It may be possible that the increased expression of genes in response to maternal vitamin C treatment in late gestation could serve as a cellular pool of resources that could be rapidly translated in the event that the function of these pathways was required to be activated in preparation for birth. As there was no effect on the numerical density of surfactant protein-producing cells present in the fetal lung, this suggests that the effects of vitamin C on surfactant maturation are regulated at the molecular level in the fetal lung. This study warrants future work to interrogate the specific cellular and translational mechanisms of antenatal antioxidant exposure on the fetal lung. While this study has focused on understanding the molecular regulation of fetal lung maturation by maternal vitamin C administration for a month in late gestation, the functional physiological consequences of vitamin C exposure on the fetal lung and surfactant function have not been assessed. Other antioxidants, such as Trolox (an analog of vitamin E), have been investigated in rats to support respiratory function in the newborn period by targeting mechanical ventilation-induced oxidative stress locally at the diaphragm.^49^ Therefore, there are many areas of interest to investigate actions of different antioxidants in the fetal, newborn, and postnatal lung, which need to explore the timing, dose, and mechanism of action.

In regards to structural lung maturation, vitamin C has previously been demonstrated to lead to increased collagen expression and extracellular matrix remodeling, a key component of large structural airway development.^50–52^ However, in this study there was no effect of maternal vitamin C treatment on a marker of collagen content. As the maternal vitamin C treatment was initiated in late gestation, past 100 days, and in the canalicular phase of fetal lung development, it is likely that the majority of large structural airway development had already occurred in the early embryonic and pseudoglandular phases. Interestingly, there was increased expression of *ELN* in the fetal lung, which is suggestive of potential alterations to small airway remodeling and/or alveolarization at the parenchymal level following exposure to maternal Vitamin C treatment.^53^ While Vitamin C has previously been shown to play a role in extracellular matrix remodeling in an emphysema model,^52^ the effects of vitamin C on proliferation and apoptosis in the fetal lung are unknown.

Since a number of fetal lung maturational signals are stimulated by glucocorticoids,^33,54^ it was important to determine possible effects on fetal plasma cortisol concentrations and on the regulation of cortisol bioactivity and availability. Additional data presented in the current study show that maternal Vitamin C treatment did not affect fetal plasma cortisol concentration in an umbilical blood sample taken at the end of the experiment. However, maternal Vitamin C treatment was associated with a fall in *HSD11B-2* expression in the fetal lung. While this finding suggests the potential for an increase in local glucocorticoid availability in the fetal lung at the gene level,^55^ there was no significant effect of maternal vitamin C on 11βHSD-2 protein expression or on the gene expression of glucocorticoid receptors (*NR2C1* and *NR2C2*) in the fetal lung.

Vitamin C was chosen for administration in this study due to it being a commonly used supplement in pregnancy with antioxidant properties.^11,56,57^ However, vitamin C is a comparatively weak antioxidant with limited capacity to compete for superoxide from reacting with nitric oxide in vivo.^58–60^ To compensate, higher concentrations of vitamin C are required. Therefore, the dose of vitamin C administered in this study was the same as that used in our previous studies validating successful antioxidant properties in the sheep fetus in vivo,^29^ and it approximated eight times that used in previous human clinical studies.^56^ Clinical studies, such as the VIP (vasoactive intestinal polypeptide) and INTAPP (international trial of antioxidants in the prevention of preeclampsia) clinical trials against preeclampsia, reported an increase in the rate of low birth weight and fetal/newborn mortality in mothers treated with vitamin C compared to those treated with placebo.^56,61^ Other studies have reported that excess vitamin C can promote kidney stones.^62^ Therefore, while data in the present study were not associated with growth restriction or fetal death and support maturational effects of maternal vitamin C administration on fetal lung maturation at the molecular level in a healthy pregnancy, we strongly agree that vitamin C is not the antioxidant of choice for translation to human therapy. Future studies should focus on antioxidants of improved human translational potential that have the greater antioxidant capacity or more targeted effects on the oxidative stress pathway at the cellular level, such as the mitochondria-targeted antioxidant MitoQ.^63^

There are important limitations to the data presented in this study. First, all fetuses in this study were male to control for, but not to address sex differences. While there is evidence that males are more vulnerable to respiratory complications at birth, our previous studies have observed no effect of sex on the expression of surfactant protein markers in the fetal lung at 133 days gestation.^38^ Second, the tissues generated in this work were part of a program of research designed with a different primary objective. This was to investigate the effect of maternal treatment with vitamin C on cardiovascular outcomes in the offspring.^27^ The fetuses did not undergo any other additional interventions as part of this primary study other than receiving either daily material administration of saline or vitamin C (105–138 days gestational age). This study used the tissues generated to begin to address additional important scientific questions retrospectively, thereby making the best use of the valuable experimental material. This scientific approach is strongly promoted by the UK Home Office 3R principle of Replacement, Reduction and Refinement and it is designed to ensure more humane animal research. Consequently, no prospective functional outcomes were performed and, in this study, lung tissue was immersion and not perfusion fixed. Therefore, a more detailed quantitative investigation of lung structure could not be performed. Nevertheless, the significant maturational effects of vitamin C on the transcriptional capacity of the fetal lung in healthy pregnancy determined in the present study, provide proof of concept that maternal supplementation with vitamin C may provide benefits in pregnancies with inadequate endogenous antioxidant protection against oxidative stress. This may include pregnancies at risk of preterm delivery with immature antioxidant defenses to withstand the relative hyperoxia of birth,^64,65^ or those with depleted antioxidant protection as a result of an increased pro-oxidant environment, such as in pregnancies complicated by preeclampsia, asthma, premature rupture of membranes, gestational diabetes, maternal smoking, or pollution exposure.^1,3,6,7,9,66,67^ Therefore, a rich avenue of research will be to design studies with a specific focus on the lung, investigating the effects of different antioxidants of greater human translational capacity on fetal lung maturation, matched with newborn and longer-term functional outcomes in healthy as well as in sub-optimal pregnancy, working towards improving respiratory outcomes in babies born from complicated pregnancies.

## Patient statement

Patient consent was not required. All procedures were approved by the animal ethics committee.
